# Obligations of the Medical Profession

**Published:** 1911-04

**Authors:** Frances Bradley


### OBLIGATIONS OF THE MEDICAL PROFESSION.

By Frances Bradley, M. D.

Please do not think from the title of my paper, that I am going to preach tonight, for I imagine most of you gentlemen get your share of lectures at home, but, surely, it is not amiss occasionally to think over our position as individuals, as citizens and as physicians. Society is dependent for its well-being and efficiency upon the Medical Profession. Even our worthy City Council grants the good health of the community as our most valuable asset and as first in importance in making up their budget. As guardians of the same we play a most important role in the economy of the municipality.

But in turn, we are indebted to the public for our clientele, for our inspiration, for our living, and in proportion to our opportunities are our obligations. If we hoard our talents, polishing them only for our own selfish advancement, we may acquire wealth, social prominence, political favor, but have we made our lives worth living? Can we at the end look back with satisfaction at the burdens we have lightened, the ways we have cleared for those not able to help themselves? The physician cannot plead that his life is of necessity one of hardship and self-sacrifice. He chooses it with his eyes open. He knows he is the servant of the people, he deserves no credit for doing his best by his patients any more than a mother deserves credit for the years of patient suffering and privation her children require. She wants the children, she gives her life to them because she loves them and because it is her business. Like the doctor she deserves no credit for doing her duty, only discredit when she fails.

The unthinking animal has no responsibility, no obligation, feels no compensation for well-doing, is allowed no choice of living a selfish or a generous life. He knows only the instincts of self-preservation. As he advances a grade in the scale of civilization, he acquires a sense of fairness, or justice, of rights of others, and finally, realizes his own and his neighbors' relative place in life. He finds that he lives and thrives and prospers at the expense of his neighbor, and in turn he is rightfully his neighbor's keeper. We each profit by the knowledge and experience of the other—and in proportion as we prosper and acquire success in life does our obligation increase to turn back that knowledge to the benefit of those less blessed. Knowledge and skill are real and tangible assets; we have acquired them as we add bonds and stocks to our bank account. We can no more carry them with us than the miser his wealth. They are ours for use but not for keeps.

Because we have the moral right to choose our life, we must accept the result of our choice be it praise or blame. The imbecile, the lunatic, the sleep-walker is exempt, from responsibility. He is un-moral, involuntarily. The moral actor must be a voluntary actor, and as such we assume the dignity of thinking, responsible beings.

It is repugnant to the self-respecting man to be badgered or coerced. We love our freedom and independence, we pride ourselves on our good intentions, our honest purposes and perhaps an occasional definite accomplishment. But if we keep as close an account of our own as of our neighbor's short-comings, if we are as keen to help our neighbor as ourselves, we shall soon see how insensibly the moral shades into the unmoral, the responsible into the irresponsible.

Huxley declares that "if some great power would agree to make him always think what is true and do what is right on condition of being turned into a sort of clock and wound up every morning he would instantly close with the offer." This may have been Mr. Huxley's idea of a compensating life, but to rob us of choice would take from us all zest of right living, and our mechanical lives lacking the voluntary element would lack all that makes them worth living. Rather would we stumble, and if necessary fall short of our ideal in the voluntary desire and effort to live worthy, efficient lives. We cannot, if we would, be passive, and it is for us to say in what direction our influence shall be felt. The physician, of all men, comes in closest contact with the home life, his word is law, his patients have faith and confidence in his skill and judgment. It is for him to say when the baby shall be weaned, and what it shall be fed, whether Mary shall go to chool at 6 or 8—whether the second case of typhoid, of tuberculosis was due to carelessness in the home or out of it. These and a thousand other questions are left implicitly with the doctor. There is no man in the community to-day who wields so powerful an influence in the growth and development of the family. His duty is not done when he treats his patient and collects his fee. The patient is an incident in a train of circumstances, which only the up-to-date physician can handle. The protection of the family and of the community is of far greater value than services rendered the patient and equally obligatory. The control of epidemics, reduction of nerve strain under which we are living, simplifying the school lives of our children, raising the standard of our public health, improving the sanitation of our homes and cities—these are things which none but the physician can accomplish. And even he cannot do it alone. Much of this work must be done by the mothers and housewives under the guidance and direction of the public-spirited physician. If the work is to be thorough and far reaching, it must be preventive, not curative, in character. In the face of many shining exceptions the Medical Profession has earned the reputation of considering it bad business to do much preventive work. This impression instigated by quacks and pseudo-scientists has gained a firm hold in the minds of the laity, and it is for us to convince them of the contrary.

We all know what has been done in the Philippines, in Cuba, and Panama, by the medical profession. We know that the latter country at least reports to-day a lower death rate than we ourselves, due entirely to up-to-date methods of administration, of hygiene and sanitation. That only an aggressive, systematic campaign on the part of the medical profession saved these countries of civilization. What, then, is the matter with giving a little attention to the needs of our own? In those far off lands we are dependent upon military discipline for results. Here it is a matter of public education. Those people had to be saved in spite of themselves, here they are clamoring for knowledge.

For the first time in the history of the world women want to know why they are losing so many of their babies, why successive cases of cancer, typhoid, tuberculosis occur in their families, why their children have adenoids, bad eyes and ears, why they are nervous, anaemic and under weight. Mothers never before wanted so much to be intelligent along the lines of hygiene and sanitation, for they never realized as now their possibilities, their responsibilities. They know nothing of the field of preventive medicine, they over-estimated the mystic power of quack remedies and profuse advice of kindly neighbors, but to-day they come to the profession, asking intelligent instruction, how to feed and clothe and house their families, how to keep them strong, sturdy and resistant. The average young mother knows no more than did her equally ignorant mother before her. For some reason mothers hesitate to tell their daughters things they have a right to know. Fathers leave their sons to gain a knowledge of themselves and of the world from any but desirable sources. A lack of confidence arises between parent and child which only perpetuates the proverbial mystery and ignorance of life. I content that the modern mother wants to be a wise mother and when she fails with her children it is due to ignorance, not indifference. Also, that the medical profession has no right to refuse her *the* help she asks—that of intelligent instruction.

The Public Health Ed. Committee of the American Medical Association believes that the salvation of the country, morally and physically, depends upon the education of the masses, that the standard of our Public Health can be raised far more than has been done in the tropics, and that to the Medical Profession will rightfully belong the credit of being the great uplifting power of the century.

With this end in view the County Medical Societies all over the country have been asked to bestir themselves not only through the municipal authorities but through the homes and schools. The former is entirely in the hands of you gentlemen, tht latter, in Atlanta, is being ably cared for by Dr. Collins. My field lies especially in the homes where I believe the battle must largely be fought and won.

Valuable, systematic education should be given in the schools, but the child belongs first of all to the parents and to them belongs the duty of instructing them as to the relation of child to parent, of origin of life, etc.

If the mothers and fathers can be educated we will have gone a long way towards reclaiming the coming generation.

Fulton County Medical Society has generously responded to the call of the public in allowing me to arrange this course of open meetings for the instruction of the mothers. Any one attending the discussion on Infant Mortality could not fail to realize the importance of the meeting nor the earnestness of the women present. They were not society women, not suffragettes, only plain, earnest mothers trying to do better work in their sphere of life. And as a result of that meeting we have had repeated requests for public health work from a dozen small towns of Georgia, from settlements and from ignorant, hard-working women unable to attend these meetings, but realizing their need of instruction.

None but physicians, members of this society, can do this work and it will take little of your time. There are to be three more dates, one every two months, and I ask you to attend these meetings and help the mothers in their efforts to raise a generation of men and women with clean, healthy minds and bodies.

